# Behavioral and physiological changes during heat stress in Corriedale ewes exposed to water deprivation

**DOI:** 10.1186/s40781-017-0140-x

**Published:** 2017-07-07

**Authors:** Jalil Ghassemi Nejad, Kyung-Il Sung

**Affiliations:** College of Animal Life Sciences, Kangwon National University, 24341 Chuncheon, Republic of Korea

**Keywords:** Behavior, Heat stress, Physiological parameters, Water deprivation, Ewes

## Abstract

This study was conducted to investigate the behavioral and physiological changes of heat stressed Corriedale ewes exposed to water deprivation. Nine Corriedale ewes (average BW = 45 ± 3.7 kg) were individually fed diets based on maintenance requirements in metabolic crates. Ewes were assigned into three groups (9 sheep per treatment) according to a 3 × 3 Latin square design for 3 periods with 21-d duration for each period. The control (CON) group was given free access to water, 2 h water deprivation (2hWD), and 3 h water deprivation (3hWD) following feeding. No differences were found in fecal excretion frequency, standing frequency (number/d), and sitting frequency among the groups (*p* > 0.05). Measurements of standing duration (min/d) and urine excretion frequency (number/d) showed a significant decrease whereas sitting duration (min/d) showed a significant increase in the 2hWD and 3hWD groups when compared with the CON group (*p* < 0.05). Fecal score and heart rate (number/min) were not different among the groups (*p* > 0.05). However, respiratory rate (number/min) and panting score were found to be significantly higher in the 2hWD and 3hWD groups than in the CON group (*p* < 0.05). It is concluded that water deprivation following feeding intensifies physiological heat stress related indicators such as respiratory rate and panting score and changes behavioral parameters such as water intake and urine excretion frequency in heat stressed ewes. Daily adaptation to the extreme environmental conditions may occur actively in ewes.

## Background

Water is the main factor for animal nutrition and any changes in its availability may lead to direct alteration in behavior of animals [1, 2]. However, ruminants, particularly sheep, are less susceptible to water scarcity than other domesticated animals [3]. Water deprivation is one of the main factors which affect ruminants’ behavior due to water availability. In arid environments, ranging animals are usually faced with water scarcity due to long distance walks in search for water. Thus, these animals have developed different behavioral and physiological adaptation mechanisms which enable them to tolerate dehydration [2, 4, 5]. Adaptation reactions represent a modification of ongoing physiological mechanisms that allow an animal to respond to stress stimuli with minimum alteration in homeostasis. Water deprivation may exacerbate the impact of heat stress and consequently the behavior of animals, especially in hot and humid climates [2, 3, 6]. It has been observed that restricting water intake in sheep [3] causes a decline in the water intake and changes drinking behavior [7]. Although the effects of adding water to TMR [8] on water intake during normal environment (comfort zone) and water restriction under heat stress conditions have been investigated in ewes [3, 4, 8] and cows [5]; however, the mechanism to which ruminants adapt themselves physiologically and behaviorally to the extreme environmental conditions need more insights. Therefore, the effects of water deprivation on behavior including sitting and standing frequency and duration, and physiological parameters including fecal score, urine and fecal excretion behavior, respiratory rate, and heart rate and possible physiological and behavioral adaptation to the extreme environmental conditions in ewes has yet to be investigated.

## Methods

### Animals and treatments

The experimental procedure and methods were approved by the Animal Welfare and Ethics Authority of Kangwon National University, Chuncheon, Republic of Korea. Nine 3-yr. old Corriedale ewes (ave. BW = 45 ± 3.7 kg), individually fed diets based on maintenance requirement in metabolic crates (0.75 m × 1.45 m), were assigned to three different treatment groups (9 ewes per treatment) according to a Latin square design (3 × 3) for 3 periods of 21-d each. The control (CON) group was given free access to water, 2 h water deprivation (2hWD), and 3 h water deprivation (3hWD) following feeding. Following the water deprivation time, ewes were given free access to water. Ewes were acclimated to the environment and experimental room conditions for 10 d prior to the experiment. Each 21 period consisted of an adaptation period (14 days) during which sheep were allowed to adapt to the new treatment group, and a measurement period (7 days) during which behavior and physiological parameters were recorded. During the experiment, temperature and humidity were controlled and maintained with little variation. The experimental room was facilitated to control temperature. Lighting in the experimental room was maintained between 08:00 and 21:30 h.

### Measurements

Feed was provided as a commercial TMR (F:C = 30:70, ~ 600 g/kg DM, ~161 g/kg CP and ~690 g/kg TDN as-fed) and was weighed and offered twice daily at 09:00 h and 18:00 h. Sheep were fed based on maintenance requirements throughout the experiment, thus no residual feed remained. Water was provided in plastic buckets and was available *ad-libitum* to the CON group; however, it was provided following feeding at 11:00 h and 20:00 h for the 2hWD group, and at 12:00 h and 21:00 h for the 3hWD group. Water intake was recorded twice daily for the final 7 days (the measurement period). Thus, sheep in the 2hWD and 3hWD groups were provided water at 2 and 3 h post-feeding, respectively.

Respiration rates were measured by counting the rate of flank movement over a 30 s period and then converted to breaths per minutes twice daily at 10:00 and 14:00. Respiratory rate was defined according to Silanikove [9]; low: 40–60 breaths/min; medium high: 60 to 80 breaths/min; high: 80 to 120 breaths/min and severe heat stress: above 200 breaths/min. Respiratory scoring was done twice daily in the morning between 10:00 to 12:00 and in the afternoon between 14:00 and 16:00 using a scale of 1 = normal, 2 = slight cough, 3 = moderate cough, 4 = moderate to severe cough, and 5 = severe and chronic cough [10, 11]. Fecal scores were assigned for each sheep twice daily at 10:30 and 15:30 using a 5-point scale. The fecal scores were assigned based on the following criteria; 1 = firm, dry, well-formed pellet; 2 = loose to firm, moist, well-formed individual pellet; 3 = soft, moist pat with some indication of individual pellet formation, 4 = soft, wet pat with no pellet formation; 5 = diarrhea. Heart rate was measured using a veterinary stethoscope twice daily during the last 7 days (measurement period). Panting score was calculated based on the scale of 0 to 4 where: 0 = no panting, 1 = Slight panting, mouth closed, 2 = Fast panting, occasional open mouth, 3 = Open mouth and some drooling, 4 = Open mouth, tongue out and drooling [10, 11].

Ewes behavior categories in this study included standing duration, standing frequency, sitting duration, sitting frequency and urine and fecal excretion frequencies that were monitored by camera every 15 min from 09:00 to 18:00 h and direct human observations using sheet scale scoring during last 5 days of the measurement period. The recording films were collected and filed in a hard disk at the end of each observation day, compared with the direct human observations, and then finalized the obtained data for statistical analysis at the end of study. Standing duration defines the average time that ewes in each group were standing until sitting. Standing frequency defines the average number of times that ewes sat and stood again during recording days. Sitting duration and frequency also define the same way as standing. Urine and fecal exertion frequency are defined the number of times that ewes excreted urine and feces, respectively.

### Temperature-humidity index (THI) in the experimental room

Housing unit temperature and relative humidity were monitored at hourly intervals, throughout the study using a temperature-humidity data logger device (CEM-DT-172, No. 11048007, Shenzhen, China). Average temperature-humidity index (THI) in the housing unit was calculated using the equation of Marai et al. [12] which defines heat stress condition.THI = db^°^C − {(0*.*31–0*.*31 RH)(db^°^C − 14*.*4)} where, db^°^C is the dry bulb temperature (°C) and RH is the relative humidity. Average THI in the experimental room was 28.9 throughout the experiment calculated using the equation of Marai et al. [12] which defines severe heat stress conditions.

### Data analysis

The focus of interest in this study had nothing to do with possible time effects on treatment mean. Therefore, we did not aim to find environmental differences in each 21 day duration since we used a controlled environmental housing system. The housing system allowed us to control temperature and humidity indoors. The interval between administering treatments helped us to minimize carry-over effects of the previous treatment. Using a Latin square design each animal received all treatments in different periods. Thus we analyzed our data using the MIXED procedure of SAS (version 9.0; SAS institute Inc., Cary, NC) for a Latin square (3 × 3) design. The model included sheep as a random effect, treatment, and period as follows: Y_ijkl_ = μ + α_i_ + S_ij_ + P_k_ + ε_ijkl_ where, Y_ijkl_ = each observation, μ = total mean, α_i_ = effect of treatment, S_ij_ = random effects of sheep × treatment, P_k_ = effects of period, and ε_ijkl_ = error. The Tukey kramer test was used for ranking treatment means and statistical differences were considered significant at *p* < 0.05. Data of THI were averaged to weekly mean (hourly interval) and analyzed as repeated measures using the MIXED procedure of SAS (version 9.0; SAS institute Inc., Cary, NC). The covariance structures (autoregressive order 1, unstructured and compound symmetry), for the repeated measures model, were tested and the structure with the best fit was chosen based on the smallest Schwarts’s Bayesian criterion.

## Results

### Temperature-humidity index, water intake and behavior

**Fig. 1:**
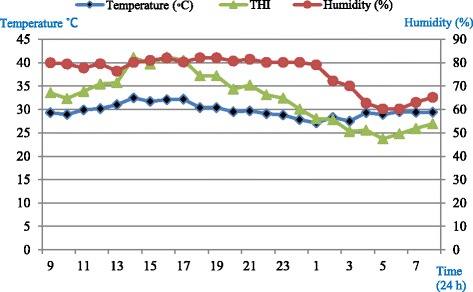
Diurnal temperature and humidity pattern in the experimental room (09:00 am – 08:00 am)

Diurnal temperature (°C) and humidity (RH%) pattern in the housing units are presented in Fig. 1.

**Fig. 2:**
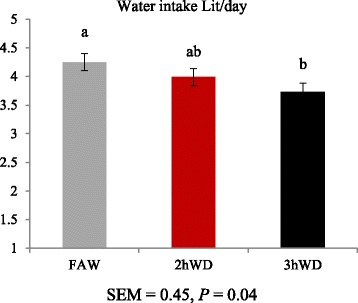
Water intake in ewes during heat stress conditions and water deprivation (*n* = 9) Bars with different superscripts differ significantly (*P* < 0.05)

Daily water intake (mL/d) tended to decrease as water deprivation time increased (*p* = 0.04, Fig. 2).

Fecal excretion frequency (number/day) was not different among treatment groups (*p* > 0.05) whereas urine excretion frequency (number/day) was higher (*p* < 0.05) in the CON group when compared to the other two treatment groups (Table 1). Standing frequency and sitting frequency were not different (*p* > 0.05) among treatment groups.

**Table 1:** Behavioral changes in sheep during heat stress conditions and water deprivation (*n* = 9)

Items	Treatments^c^			SEM	*P* - value
	CON	2hWD	3hWD		
Urine excretion frequency (number/day)	10.4^a^	6.9^b^	6.9^b^	1.17	0.03
Fecal excretion frequency (number/day)	3.7	4.0	4.2	0.27	0.28
Standing frequency (number/day)	19.1	20.8	22.3	1.43	0.54
Sitting frequency (number/day)	18.2	19.9	20.8	2.07	0.68
Standing duration (min/day)	317.9^a^	275.2^b^	273.4^b^	17.41	0.03
Sitting duration (min/day)	405.1^b^	446.0^a^	449.4^a^	17.71	0.03

^a, b^Values within a row with different superscripts differ significantly at *P* < 0.05

^c^Treatments included free access to water (CON), 2 (2hWD) and 3 h (3hWD) water deprivation following feeding

In the 2hWD and 3hWD groups standing duration (min/d) decreased and sitting duration (min/d) increased significantly when compared to the CON group (*p* < 0.05, Table 1).

### Physiological parameters

Fecal score and heart rate (number/min) were not different (*p* > 0.05) among treatment groups (Table 2). Both respiratory rate (number/min) and panting score showed no significant differences (*p* > 0.05) among treatment groups for measurements in the afternoon. In contrast, in the morning measurements, respiratory rate and panting score were higher (*p* < 0.05) in sheep in water deprived groups than the CON group. Total daily respiratory rate (number/min) and total daily panting score were higher (*p* < 0.05) in the 2hWD and 3hWD groups than in the CON group (Table 2).

**Table 2:** Physiological parameters in ewes during heat stress conditions and water deprivation (*n* = 9)

Items	Treatments^c^			SEM	*P* - value
	CON	2hWD	3hWD		
Respiratory rate (am,number/min)	81.5^b^	113.5^a^	113.6^a^	3.24	0.03
Respiratory rate (pm,number/min)	96.7	96.3	97.9	3.24	0.32
Respiratory rate (total,number/min)	88.8^b^	107.2^a^	106.1^a^	2.94	0.02
Heart rate (am,number/min)	72.0	69.9	72.8	1.99	0.41
Heart rate (pm,number/min)	60.0	62.1	62.1	1.19	0.37
Heart rate (total,number/min)	66.0	66.0	67.5	1.19	0.59
Panting score (am)	1.4^b^	2.4^a^	2.1^a^	0.09	0.04
Panting score (pm)	1.6	1.4	1.8	0.09	0.13
Panting score (total)	1.5^b^	1.9^a^	1.9^a^	0.08	0.03
Fecal score	1.3	1.3	1.2	0.05	0.51

^a, b^Values within a row with different superscripts differ significantly at *P* < 0.05

^c^Treatments included free access to water (CON), 2 (2hWD) and 3 h (3hWD) water deprivation following feeding

## Discussion

Water is lost via urination, defecation, and evaporation from skin and respiratory tract [1, 3, 13]. High ambient temperature may exacerbate body water loss. When sheep are exposed to increased ambient temperature, water intake is expected to be increased [14, 15], and it is expected that water restriction may increase water intake under hot conditions. Ruminants are able to conserve water with a reservoir system for use in periods of reduced water supply [1, 15]. The aforementioned reports may explain why sheep in the 3hWD showed less water intake than those in the CON group.

Sheep are especially well adapted to produce concentrated urine [15, 16] and, in particular, less urine in volume when water is a restricted factor [13, 15]. This ability of sheep to produce highly concentrated urine enables sheep to have a high tolerance to hot conditions with limited water availability [3, 13, 17]. This and the fact that sheep in water deprived groups showed lower water intake may explain the less number of urination in sheep exposed to water deprivation compared with the CON group.

Ruminants are active during the day and rest during the night. However, under heat stress condition, grazing ruminants tend to lie down to reduce their locomotion during the day [9, 18]. Adaptation and acclimatization favor sheep to cope with hot and humid environment especially when water is restricted. In this study, sheep in water-deprived groups showed an elevation in sitting duration and decline in standing duration which was counter to our hypothesis. Many studies reported that ruminants tend to stand more in the barn while exposed to heat stress in order to reduce their body temperature by exposing body surface to wind [9, 12]. However, a possible explanation for these phenomena is that standing consumes more energy. Sheep in this study were fed based on maintenance requirements and were therefore kept in individual crates in a housing unit (no wind). In addition, sheep had over 3 cm fleece which results in maintaining heat around the body compared to cattle and goats. Unlike several studies [9, 12] that reported higher standing time during heat stress, in the present study, ewes were kept in metabolic crates under confined housing conditions; thus, no wind circulation existed. This and what we have discussed earlier may explain why ewes in this experiment showed lower standing duration when the heat stress condition exacerbate by exposing to water deprivation.

The general homeostatic responses to heat stress in ruminants include, but are not limited to; raising respiration rates [19], panting, and reduce heart rate as revealed in this experiment. Normal resting respiratory rate for sheep has been reported [17, 20, 21] to be between 20 and 38 breaths/min. However, it is demonstrated that the resting respiratory rate of sheep to be increased considerably if the animals are excited [20]. Panting score has been used as the easiest method of evaluating the impact of heat stress. This is because it only requires direct observation of the animal. Panting is known as sheep’s response to increased environmental heat; by substantial increasing of respiratory rate. There are two phases of panting in sheep [20]; rapid shallow panting, and the slower deeper panting. An increase in both first and second phase panting is highly correlated with increasing ambient temperature and humidity [19, 21]. Increasing respiratory rates are the first sign of panting [20]; however, respiratory rate varies between individuals [17, 19]. Stockman [11] showed that there is a relationship between sheep behavior and panting as panting can be differentiated by several observable behavioral changes. The cessation of panting during rumination prevents major heat loss. Physiological changes include changes to respiratory rate, heart rate, and panting score. These are main visual indicators of heat stress showed higher values in the morning than in the afternoon meaning that sheep adapt themselves daily to the hot climate and water deprivation. Additionally, it is documented that ruminants are able to adapt themselves quickly to the environmental conditions. As it can be clearly seen (Fig. 1), the temperature, humidity, and THI dropped down to a comfort zone (no heat stress conditions) at night between 12 midnight until 7 am and then again routinely began to go up gradually. Moreover, any adaptation in physiological parameters should start in metabolic level. It is assumed that the metabolic adaptation by means of having free access to water (for 2hWD and 3hWD groups, 2 and 3 h post-feeding respectively) could help the animals by cooling down and also dissipating heat from the body. In this study, ewes were offered feed two times in the morning at 09:00 and 18:00 based on maintenance requirements. Thus, gastro-intestinal (GI) movements that help internal heat (heat increment) were actively available post-feeding. This and the fact that ewes had free access to water maximum 2 (in 2hWD group) and 3 h (in 3hWD group) following morning feeding at 09:00 until evening feeding at 18:00, help us to understand that the THI is not the only factor that can affect physiological parameters such as respiratory rate and panting score. Feeding may have synergist effects on body core temperature and consequently, increase the potential of heat stress related indicators when drinking water can help the animals to cool down their body. The aforementioned reasons altogether may explain why panting score was lower in the afternoon while the THI was a bit higher. Despite the fact that long-term acclimation to hot and humid conditions for ruminants is well documented [3, 17, 18], from these results it may suggest that adaptation to heat stress conditions and water deprivation is an active daily response.

Being exposed to heat stress changes blood flow in the body of sheep [22] which may result in the most obvious physiological effect of heat stress by an increase in heart rate [11]. However, no increase in heart rate of sheep in response to increased heat stress was observed in another study [23].

## Conclusion

Authors conclude that water deprivation following feeding decreases water intake, intensifies physiological heat stress related indicators include respiratory rate and panting score, and decrease water intake and urine excretion in heat stressed ewes. Higher values of respiratory rate, heart rate, and panting score in the morning than in the afternoon may imply that sheep adapt themselves daily to the hot climate and water deprivation; however, more research is needed to validate this result.

## Abbreviations

2hWD: 2 h water deprivation
3hWD: 3 h water deprivation
BW: Body weight
CON: Control
CP: Crude protein
DM: Dry matter
F:C: Forage to concentrate ratio
H: Hour
Kg: Kilogram
Lit: Litter
Min: Minute
TDN: Total digestible nutrients
THI: Temperature-humidity index
